# Determinants of patient activation and its association with cardiovascular disease risk in chronic kidney disease: A cross‐sectional study

**DOI:** 10.1111/hex.13225

**Published:** 2021-04-09

**Authors:** Thomas J. Wilkinson, Katherine Memory, Courtney J. Lightfoot, Jared Palmer, Alice C. Smith

**Affiliations:** ^1^ Leicester Kidney Lifestyle Team Department of Health Sciences University of Leicester Leicester UK

**Keywords:** cardiovascular disease, chronic kidney disease, cross‐sectional, multimorbidity, patient activation, self‐management

## Abstract

**Background:**

Patient activation describes the knowledge, skills and confidence in managing one's own health. Promoting patient activation is being prioritized to reduce costs and adverse outcomes such as cardiovascular disease (CVD). The increasing prevalence of chronic kidney disease (CKD) presents a need to understand the characteristics that influence patient activation and the effect on health outcomes.

**Design:**

Cross‐sectional study.

**Setting and participants:**

Patients with non‐dialysis CKD recruited from 14 sites (general nephrology and primary care) in England, UK.

**Outcome measures:**

Patient activation was measured using the PAM‐13. Demographic and health‐related variables, self‐reported symptom burden, health‐related quality of life (HRQOL), socioeconomic status (SES), were assessed as determinants of patient activation. Major CVD risk factors included hypertension, dyslipidaemia, obesity and hyperkalaemia.

**Results:**

743 patients were included (eGFR: 32.3 (_SD_17.1) mL/min/1.73 m^2^, age 67.8 (_SD_13.9) years, 68% male). The mean PAM score was 55.1 (_SD_14.4)/100. Most patients (60%) had low activation. Those with low activation were older (*P*<.001), had lower eGFR (*P* = .004), greater number of comorbidities (*P* = .026) and lower haemoglobin (*P* = .025). Patients with low activation had a 17% greater number of CVD risk factors (*P* < .001). Risk factors in those with low activation were being older (*P* < .001) and having diabetes (*P* < .001).

**Conclusion:**

This study showed that only a minority of CKD patients are activated for self‐management. Our findings help better understand the level of activation in these patients, particularly older individuals with multimorbidity, and further the knowledge regarding the characteristics that influence activation.

**Patient or Public Contribution:**

Patients were involved in the design of main study.

## 1 Introduction

In a growing multi‐morbid population, managing the demand for health services is a challenge faced by physicians and policymakers. In the UK, 70% of National Health Service (NHS) expenditure is spent on patients with long‐term conditions (LTCs) such as chronic kidney disease (CKD).^1^ With <1% of time spent in contact with health‐care professionals, many patients are expected to self‐manage their condition^1^ particularly those with less advanced disease. The importance of self‐management is recognized in the NHS Universal Personalised Care and Long‐Term plans,^2^ and self‐management is integral to any model of care for LTCs.^3^, ^4^ ‘Patient activation’ describes the knowledge, skills and confidence a person has in managing their own health.^5^ The ‘Patient Activation Measure’ (PAM) is the most widely used instrument for measuring patient activation^6^ and was piloted in the NHS through the UK Renal Registry (UKRR). Increased patient activation is associated with improved health outcomes in many LTCs including premature mortality and hospitalization.^7^, ^8^ Patient activation is closely related to the engagement of preventive health behaviours with empirical studies indicating activated patients are more likely to attend screenings, check‐ups and immunizations, as well as engage in healthy behaviours such as eating a balanced diet.^9^, ^10^

Few studies have administered the PAM to patients with kidney disease, and as such, information regarding the determinants and outcomes in this population is sparse.^11^ Nonetheless, promoting patient activation in kidney disease care is increasingly being prioritized and has recently emerged as central to legislative policy in the United States^11^ and UK.^12^ Studies in CKD have largely taken place in Europe,^13^ the USA,^14^, ^15^, ^16^, ^17^ Australia ^18^, ^19^, ^20^, ^21^ and Asia^22^ and often report associations between patient activation and clinical characteristics. In general, lower activation levels are associated with older age, receiving in‐center haemodialysis, poorer perceptions of health‐related quality of life (HRQOL), higher decisional conflict with respect to modality choice and lower medication adherence (summarized in Nair and Cavanuagh^11^). In the UK, Hamilton et al^23^ found higher patient activation was associated with better medication use, a younger age of renal replacement therapy (RRT) in 590 dialysis and kidney transplant recipients. Data collected as part of the UKRR ^12^ found higher patient activation levels in younger individuals and those with a kidney transplant, although no associations between patient activation and clinical biomarkers were found. Nonetheless, these findings are limited as data were almost exclusively collected in those on RRT with eGFR data missing for all of the non‐dialysis group.

The increasing prevalence of CKD presents an urgent need to understand the role of patient activation.^15^ Further evidence is also needed to show higher levels of patient activation are associated with clinically meaningful outcomes.^11^ Cardiovascular disease (CVD) remains a leading cause of premature mortality and morbidity in CKD,^24^ and reducing CVD remains a mainstay of conventional CKD management. Patient activation may be a fundamental component that can mediate the presence of CVD risk factors.

The aim of this study was to (a) explore the prevalence of patient activation across a range of adult CKD participants, (b) identify factors associated with patient activation and (c) explore the association with CVD risk factors.

## 2 METHODS

### 2.1 Study design and setting

This analysis consists of PAM data taken from the multi‐center observational DIMENSION‐KD study (ISRCTN ref: ISRCTN84422148). Participants completed a self‐administered paper survey made up of different questionnaires designed to assess physical function and activity, symptoms and diet. Participants were recruited from general nephrology clinics and from GP practices between July 2018 and February 2020 across 14 sites (all sites recruited from general nephrology clinics, whilst one site (University Hospitals of Leicester NHS Trust) also recruited from 8 local GP practices) in England, UK (supplementary material 1)). Participants were included if they had been (a) diagnosed with a kidney condition (CKD 1‐5 not requiring dialysis), (b) were aged ≥ 18 years and (c) were able to provide informed consent. Participants with or receiving RRT (dialysis, transplant) were excluded. The study was granted national research ethical approval (18/EM/0117). All patients provided informed written consent, and the study was conducted in accordance with the Declaration of Helsinki.

### 2.2 Patient activation measure‐13 (PAM‐13)

The PAM‐13 is a validated tool of 13 questions which assesses a patient's knowledge, skills and confidence in managing their own health. The PAM‐13 has demonstrated good internal consistency as well as adequate reliability and validity.^5^, ^6^ Answers are weighted and combined to provide a score on a scale from 0 to 100. A score is generated where participants have answered ≥10 questions. The PAM allows respondents to be categorized into one of four levels with lower levels indicating low activation and higher levels indicating high activation: Level 1 (<47.0), disengagement and disbelief about one's own role in self‐management; Level 2 (47.1‐55.1), increasing awareness, confidence and knowledge in self‐management tasks; Level 3 (55.2‐67), readiness and taking action; and Level 4 (>67.1), sustainment.

### 2.3 Determinants of patient activation variables

#### 2.3.1 Demographic and clinical variables

Demographic (age, sex, ethnicity) and health‐related variables (comorbidities, smoking status, body mass index (BMI)) were self‐reported. Upon receipt of the survey, participant's most recent clinical data, including recent renal function (estimated glomerular filtration rate, eGFR), cause of disease, haemoglobin, potassium, albumin, C‐reactive protein (CRP), lipid profile, and blood pressure, were extracted from the medical records by the research team at each site.

#### 2.3.2 Symptom burden

Symptom burden was self‐reported using the Kidney Symptom Questionnaire,^25^ a validated questionnaire surveying 13 commonly reported symptoms. Patients rated the frequency and importance of each symptom on a 5‐point Likert scale (from 0 (never/not intrusive) to 4 (every day/extremely intrusive)). Total symptom burden was determined by combining the frequency (/52) and importance (/52) score to give a total score /104.

#### 2.3.3 Quality of life

Self‐reported HRQOL was assessed using the SF‐12 questionnaire, a validated assessment of quality of life that provides a physical (PCS) and mental (MCS) component score. The final score of the SF‐12 ranges from 0‐100, where a higher score indicated a better HRQOL. A score below or above 50 indicates a, respectively, worse or better quality of life than a pre‐defined general population reference group. The SF‐12 has good agreement with other measures of HRQOL including the KDQOL‐36.^26^

#### 2.3.4 Cardiorespiratory fitness

Cardiorespiratory fitness was estimated from the Duke Activity Status Index (DASI) questionnaire, a brief 12‐item questionnaire assessing the capability to complete activities of daily living (ADLs). Each activity is weighted with a metabolic equivalent of tasks value which is summed to produce a score (0 to 58.2). This was transformed into VO_2peak_ using a previously published equation.^27^

#### 2.3.5 Dietary intake assessment

Self‐reported dietary intake was assessed using the European Prospective Investigation of Cancer in Norfolk Food Frequency Questionnaire (FFQ). It has been validated against diet diaries and biological markers and widely used in the UK general population.^28^ The FFQ measured participant's food intake during the previous year. For each food item, participants were asked to indicate their usual consumption from nine categories ranging from never or <1/month to ≥6 times per day. The FFQ was used to extract fruit and vegetable intake (g/day) and alcohol intake (g/day).

#### 2.3.6 Socioeconomic status (SES)

Socioeconomic status (SES) was measured using the Index of Multiple Deprivation (IMD). It is comprised of seven distinct domains of deprivation which, when combined and appropriately weighted, form the IMD score. Income (22.5%) and employment (22.5%) make up the two largest components of an area's IMD score. An individual's IMD was calculated from their postcode. An IMD ranges from 1 (most deprived area) to 32,844 (least deprived area). IMD deciles are calculated by ranking all 32,844 neighbourhoods in England from most to least deprived, before dividing them into 10 groups; decile 1 is the most deprived, decile 10 is the least deprived.

### 2.4 Cardiovascular disease risk factors

Major CVD risk factors were defined as either traditional [(1) age (older than median total sample 71.0 years), (2) male sex, (3) current smoker, (4) excessive alcohol intake, (>14.3 g/day); (5) hypertension (systolic blood pressure ≥ 130 mm Hg or diastolic ≥ 85 mm Hg, (6) diabetes, (7) dyslipidaemia (TG ≥ 150 mg/dL (8.3 mmol/L) or low level of HDL < 40 mg/d (2.2 mmol/L) in men and <50 (2.8 mmol/L) in women, or high LDL ≥ 100 mg/dl (5.6 mmol/L)) or high serum total cholesterol (TC) at ≥200 mg/dL (11.1 mmol/L), (8) obesity (BMI ≥ 30kg/h^2^)] and non‐traditional [(9) CRP > 4 mg/dL, (10) hyperkalaemia (serum potassium ≥ 5.0 mEq/L) and (11) anaemia (Hb < 11g/dL)].

### 2.5 Statistical analysis

Descriptive and frequency data are presented as mean (standard deviation (SD)) or number (percentage). Statistical testing was conducted using IBM SPSS V26, and a *P*‐value of <.050 was considered significant. eGFR was calculated using the EPI formula.^29^ Activation levels were dichotomized into low activation (Levels 1 and 2) and high activation (Levels 3 and 4) as reported previously.^19^ Differences between those with low and high activation were explored using general univariate models or chi‐square testing. A multivariable linear model was used to assess the association between PAM‐13 score and the following variables: age, eGFR, sex, BMI, ethnicity, IMD, number of comorbidities, symptom burden and number of medications. These variables were pragmatically chosen a priori based on hypothesized associations but also to maintain the largest sample size. Binominal logistic regression, adjusted for eGFR, was used to explore the association between different CVD risk factors and having low activation. Missing data were handled using pairwise deletion to maximize all data available per analysis. Due to missing data, CRP and dyslipidaemia were excluded to maintain a sample of 340. Data are shown as odds ratio with 95% confidence intervals. Missing data for each variable can be found in Table 1. Differences between groups in Table 1 were assessed using chi‐square testing or univariate regression with disease stage as the independent variable; significant ß was identified as *P* < .050, as appropriate.

**TABLE 1:** Participant characteristics

	Stage 1‐2 (n = 56)	Stage 3 (n = 291)	Stage 4‐5 (n = 396)	Total (n = 743)	Missing, n (%)	P
Age (years)	58.9 (_SD_17.2)	64.1 (_SD_12.3)	71.9 (_SD_13.1)	67.8 (_SD_13.9)	6 (<1%)	<.001^a^
Sex (male), n (%)	33 (59%)	187 (64%)	283 (71%)	503 (68%)	4 (<1%)	.010^a^
Ethnicity, n (%)						
White British	54 (97%)	272 (93%)	375 (95%)	770 (94%)	14 (2%)	.302
South Asian	1 (2%)	11 (4%)	5 (1%)	17 (2%)		
Other	1 (2%)	13 (4%)	18 (5%)	32 (4%)		
eGFR (ml/min/1.73m^2^)	74.0 (_SD_11.0)	40.1 (_SD_8.1)	20.3 (_SD_6.4)	32.3 (_SD_17.1)	0 (0%)	<.001^a^
Albumin (g/L)	40.0 (_SD_5.5)	41.3 (_SD_4.1)	39.9 (_SD_4.5)	40.4 (_SD_4.5)	236 (32%)	.044^a^
C‐reactive protein (mg/L)	13.7 (_SD_21.0)	10.1 (_SD_14.1)	10.6 (_SD_16.4)	10.7 (_SD_16.0)	580 (78%)	.682
Haemoglobin (g/L)	132.2 (_SD_22.2)	129.2 (_SD_19.1)	116.7 (_SD_19.6)	122.5 (_SD_20.8)	203 (27%)	<.001^a^
Cholesterol, total (mmol/L)	4.8 (_SD_1.4)	4.5 (_SD_1.2)	4.2 (_SD_1.0)	4.4 (_SD_1.1)	615 (83%)	.021
BMI (kg/m^2^)	28.8 (_SD_8.2)	30.3 (_SD_7.4)	28.9 (_SD_7.3)	29.4 (_SD_7.4)	151 (20%)	.439
No. of comorbidities	3.5 (_SD_1.7)	3.1 (_SD_1.7)	3.4 (_SD_1.8)	3.3 (_SD_1.7)	33 (4%)	.102
Hypertension, n (%)	19 (34%)	75 (26%)	169 (43%)	263 (35%)	284 (38%)	.007^a^
Diabetes, n (%)	9 (16%)	100 (34%)	176 (44%)	285 (38%)	20 (3%)	<.001^a^
Index of Multiple Deprivation (IMD)						
IMD score	18 362.0 (_SD_8575.0)	17 721.0 (_SD_8928.0)	18 769.2 (_SD_8252.9)	18 327.7 (_SD_8551.6)	43 (6%)	.232
Decile 1, n (%)	2 (4%)	19 (7%)	16 (4%)	37 (5%)		
Decile 10, n (%)	611%)	3 (12%)	46 (12%)	86 (12%)		

Note.

Data presented as mean and standard deviation (SD) or number (percentage)

Abbreviations: eGFR, Estimated glomerular filtration rate; BMI, Body mass index

Stage 1‐2 defined as an eGFR of ≥60 mL/min/1.73 m^2^; Stage 3 as 59‐30 mL/min/1.73 m^2^; Stage 4‐5 < 30 mL/min/1.73 m^2^

Statistical significance recognized as *P* < .050 and denoted by ^a^

## 3 RESULTS

### 3.1 Summary of participant characteristics

743 patients were included, the majority (92%) in Stage 3 and 4‐5, with a mean eGFR of 32.3 (_SD_17.1) mL/min/1.73 m^2^. The mean age for the cohort was 67.8 (_SD_13.9) years, and 68% were male. Most patients were White British (94%). The population were largely overweight with a BMI of 29.4 (_SD_7.4) kg/m^2^ and were comorbid with a mean additional number of comorbidities of 3.3 (_SD_1.7) (shown in Table 1).

### 3.2 Patient activation status across disease stages

**FIGURE 1:**
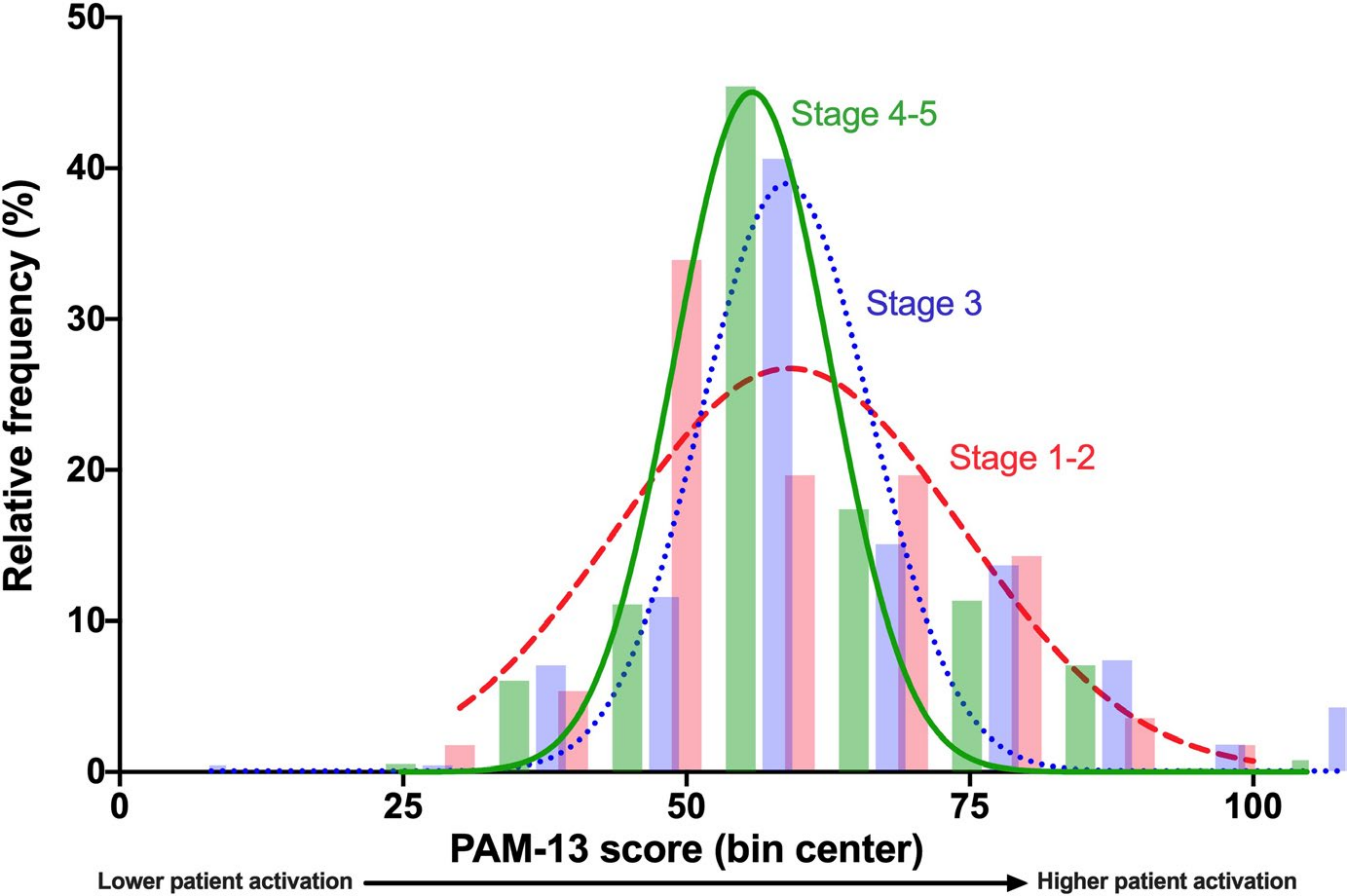
Frequency distribution curves for each disease stage. PAM, Patient Activation Measure. Data shown as relative frequency as a percentage (%)

The distribution of PAM‐13 scores across disease stage is shown in Table 2 and Figure 1. The mean PAM‐13 score was 55.1 (_SD_14.4) **out of 100**. The median PAM‐13 level was 2. Most patients (60%) had low activation levels (Level 1 and 2). PAM‐13 scores declined from 61.6 (_SD_14.6) in Stage 1‐2 down to 56.0 (_SD_16.1) in Stage 3, and 53.5 (_SD_12.7) in Stage 4‐5.

**TABLE 2:** Distribution of patient activation measure levels and scores across disease stage

Level	Stage 1‐2 (n = 56)		Stage 3 (n = 291)		Stage 4‐5 (n = 396)		Total (n = 743)	
	Mean (SD)	n (%)	Mean (SD)	n (%)	Mean (SD)	n (%)	Mean (SD)	n (%)
1 (least activated)	41.4 (_SD_ 6.3)	7 (13%)	39.5 (_SD_ 7.4)	80 (27%)	39.5 (_SD_ 6.6)	108 (27%)	39.5 (_SD_ 6.7)	195 (26%)
2	50.9 (_SD_ 1.2)	16 (29%)	51.1 (_SD_ 1.4)	91 (31%)	51.1 (_SD_ 1.4)	142 (36%)	51.1 (_SD_ 1.4)	249 (34%)
3	63.7 (_SD_ 4.2)	19 (52%)	62.7 (_SD_ 4.5)	76 (26%)	61.7 (_SD_ 4.8)	109 (28%)	62.2 (_SD_ 4.6)	204 (28%)
4 (most activated)	81.3 (_SD_ 8.1)	14 (25%)	85.2 (_SD_ 10.5)	44 (15%)	79.3 (_SD_ 7.6)	37 (9%)	82.4 (_SD_ 9.4)	95 (13%)

Note.

Data presented mean and standard deviation (SD) and number (%) for each disease stage.

Level 1 defined as PAM score of 47.0 or lower; Level 2:47.1 to 55.1; Level 3:55.2 to 67.0; Level 4:67.1 or above

### 3.3 Determinants of patient activation status

Table 3 shows differences in characteristics between those with low and high PAM‐13 levels. Low activated patients were older (*P* < .001), had a lower eGFR (*P* = .004), had a greater number of comorbidities (*P* = .026) and had lower haemoglobin levels (*P* = .025). There was no difference in other variables including IMD score. Low activation was associated with a 22% reduction in cardiorespiratory fitness compared to those with higher activation (*P* < .001). Those with low activation had a 17% lower PCS (*P* < .001) and 6% lower MCS on the SF‐12 (*P* = .005). There was no difference in fruit and vegetable intake (*P* = .457).

**TABLE 3:** Differences in characteristics between those with low and high patient activation measure levels

PAM score	Low PAM (n = 444)	High PAM (n = 299)	*P*
Age (years)	70.2 (_SD_12.8)	64.4 (_SD_14.2)	<.001^a^
eGFR (ml/min/1.73m^2^)	30.5 (_SD_15.2)	34.2 (_SD_18.8)	.004^a^
BMI (kg/m^2^)	29.4 (_SD_8.3)	29.2 (_SD_7.7)	.820
Ethnicity (% White British)	417/439 (95%)	274/294 (93%)	.176
Index of Multiple Deprivation	18 567.7 (_SD_8694.6)	17 648.0 (_SD_8447.6)	.165
Mean IMD decile	6.1 (_SD_2.6)	5.9 (_SD_2.6)	.168
No. of comorbidities	3.4 (_SD_1.8)	3.1 (_SD_1.7)	.026^a^
Albumin (g/L)	40.0 (_SD_4.7)	40.7 (_SD_4.3)	.076
C‐reactive protein (mg/L)	11.2 (_SD_16.4)	10.6 (_SD_17.1)	.773
Haemoglobin (g/L)	120.6 (_SD_20.8)	125.2 (_SD_20.5)	.025^a^
Cholesterol, total (mmol/L)	4.3 (_SD_1.2)	4.4 (_SD_1.2)	.908
Symptom burden	43.7 (_SD_21.8)	46.2 (_SD_23.8)	.248
No. of medications	7.1 (_SD_5.6)	6.5 (_SD_4.7)	.131
Fruit & vegetable intake (g/d)	436.1 (_SD_297.4)	451.8 (_SD_251.0)	.457
Cardiorespiratory fitness (VO_2peak_)	21.1 (_SD_6.6)	25.8 (_SD_6.8)	<.001^a^
PCS (SF‐12)	38.3 (_SD_11.3)	45.0 (_SD_11.3)	<.001^a^
MCS (SF‐12)	47.4 (_SD_11.1)	50.1 (_SD_9.8)	.005^a^

Note.

Data presented as mean and standard deviation (SD) unless otherwise stated.

Abbreviations: PAM, Patient Activation Measure; eGFR, Estimated glomerular filtration rate; BMI, Body mass index; PCS, Physical component score; MCS, Mental component score

Most deprived area has Index of Multiple Deprivation (IMD) rank of 1; a rank of 32,844 = least deprived area

Symptom burden defined as combined frequency and impact score from Kidney Symptom Questionnaire

Statistical significance recognized as *P* < .050 and denoted by^a^

**FIGURE 2:**
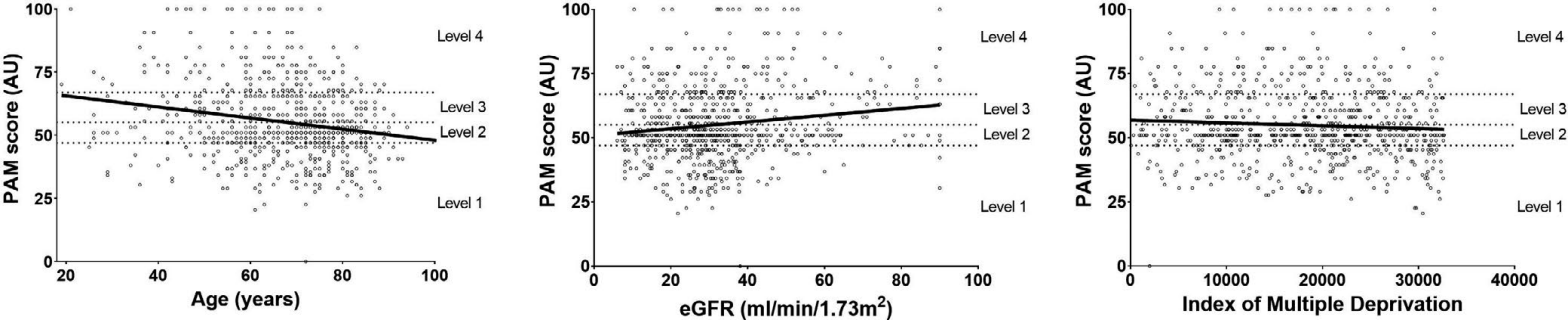
Association of Patient Activation Measure score with age, eGFR and Index of Multiple Deprivation. PAM, Patient Activation Measure; eGFR, Estimated glomerular filtration rate; AU, Arbitrary unit Level 1 defined as PAM score of 47.0 or lower; Level 2:47.1 to 55.1; Level 3:55.2 to 67.0; Level 4:67.1 or above. Data showing Patient Activation Measure score and number with low activation stratified by Index of Multiple Deprivation can be found in the online supplementary material 2

In a multivariate model, age, eGFR and IMD explained 27% of PAM‐13 score. Higher PAM‐13 scores were associated with a lower age (β = −0.207, *P* < .001), higher eGFR (β = 0.116, *P* = .007) and a lower IMD (β = −0.100, *P* = .017). For every decade increase in age, PAM‐13 score declined by 2.1, and for every 10 mL/min/1.73 m^2^ reduction in eGFR, the PAM‐13 score declined by 1.0 (Table 4). Scatter plots of the association between PAM‐13 score with age, eGFR and IMD are shown in Figure 2.

**TABLE 4:** Factors associated with patient activation measure score

(n = 548)	β	St. β	t	*P*
Age	−0.207	−0.193	−4.368	<.001^a^
eGFR	0.101	0.116	2.694	.007^a^
Sex	−0.270	−0.009	−0.223	.823
BMI	−0.122	−0.066	−1.570	.117
Ethnicity	2.162	0.059	1.415	.158
Index of Multiple Deprivation	0.000	−0.100	−2.398	.017^a^
No. of comorbidities	−0.130	−0.016	−0.391	.696
Symptom burden	0.016	0.025	0.581	.561
No. of medications	−0.071	−0.025	−0.586	.558
Constant	70.410	‐	12.503	‐

Note.

Data presented as standardized (st.) beta β and t value.

Abbreviations: eGFR, Estimated glomerular filtration rate; BMI, Body mass index

Education status defined as completion of university, college, high school or vocational qualification; symptom burden defined as combined frequency and impact score from Kidney Symptom Questionnaire

Statistical significance recognized as *P* < .050 and denoted by ^a^

### 3.4 Association between patient activation and CVD risk factors

**FIGURE 3:**
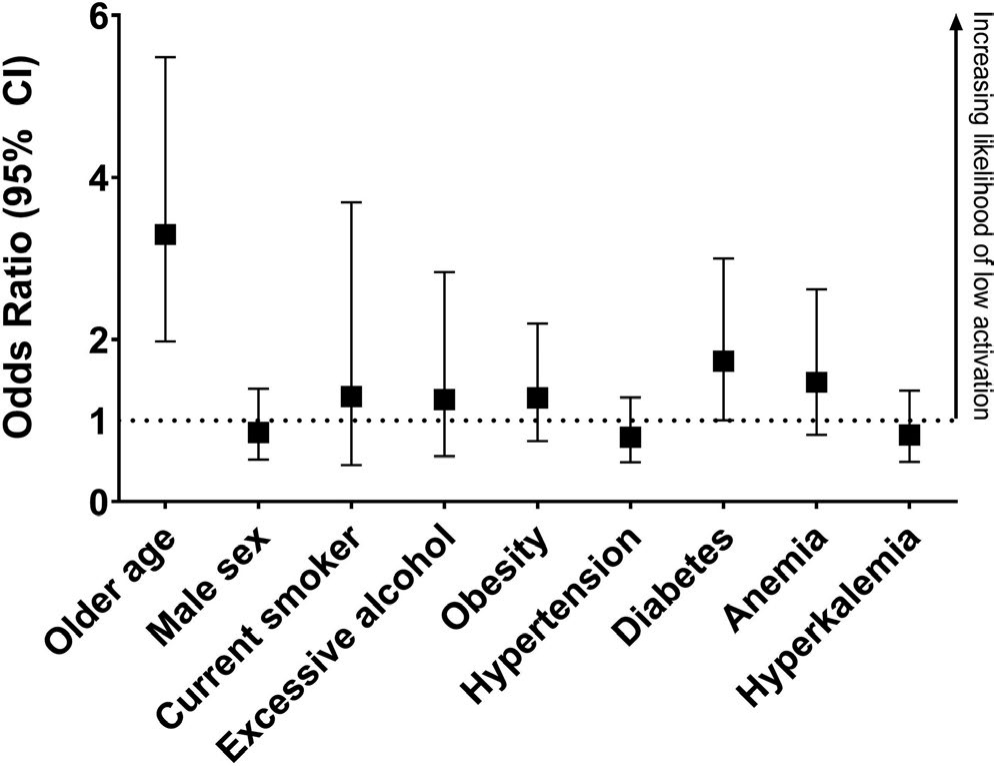
Odds of having low activation across CVD risk factors. Due to the large number of missing data, CRP, dyslipidaemia, to leave a sample of 340. Analysis adjusted for eGFR. Data shown as odds ration with 95% confidence intervals (CI). Data used in this figure can be found in the online supplementary material 3

Low activated patients had a 17% greater number of CVD risk factors than those with high activation (*P* < .001) with a greater number of patients demonstrating ≥5 CVD risk factors (*P* = .007). Significant risk factors found in those with low activation were being older (*P* < .001) and having diabetes (*P* < .001) (Table 5). Results of the logistic regression model showed that those older than 71 years were 3 times likely to have low activation (OR = 3.295, *P* < .001) and those with diabetes were 1.7 times more likely to have low activation (OR = 1.735, *P* = .049) (Figure 3).

**TABLE 5:** Differences in cardiovascular disease risk factors between those with low and high Patient Activation Measure levels

CVD risk factors	Low PAM (n = 444)	High PAM (n = 299)	*P*
Older age, >71 years old	250/444 (56%)	100/297 (34%)	<.001^a^
Male sex	267/444 (60%)	177/297 (60%)	.883
Current smoker	26/444 (6%)	11/299 (4%)	.181
Excessive alcohol, >14.3g/d	34/434 (8%)	25/265 (9%)	.705
Obesity, BMI ≥ 30kg/h^2^	143/373 (4%)	77/239 (3%)	.124
Dyslipidaemia, present†	65/108 (60%)	42/87 (48%)	.097
Hypertension, present‡	142/291 (49%)	96/198 (48%)	.946
Diabetes, present	177/431 (41%)	76/296 (27%)	<.001^a^
Anaemia, haemoglobin < 11g/dl	83/237 (35%)	47/237 (20%)	.092
Inflammation, CRP > 4 mg/dL	70/133 (53%)	43/101 (43%)	.127
Hyperkalaemia, > 5.0mEq/L	92/328 (28%)	67/247 (27%)	.806
Total no. of CVD risk factors/11	3.0 (_SD_1.6)	2.5 (_SD_1.6)	<.001^a^
5 or more CVD risk factors	87/444 (20%)	36/299 (12%)	.007^a^

Note.

Data presented as mean and standard deviation unless otherwise stated.

Abbreviation: BMI, Body mass index

Major cardiovascular disease (CVD) risk factors were defined as either traditional [(1) age (older than median total sample 71.0 years), (2) male sex, (3) current smoker, (4) excessive alcohol intake, (>14.3g/day); (5) hypertension (systolic BP ≥ 130 mm Hg or diastolic blood pressure ≥ 85 mm Hg, (6) diabetes, (7) dyslipidaemia (TG ≥ 150 mg/dl (8.3 mmol/L) or low level of HDL < 40mg/d (2.2 mmol/L) in men and < 50 (2.8 mmol/L) in women, or high LDL ≥ 100 mg/dl (5.6 mmol/L)) or high serum total cholesterol (TC) at ≥ 200mg/dL (11.1 mmol/L), (8) obesity (BMI ≥ 30kg/h^2^)] and non‐traditional [(9) CRP > 4 mg/dL, (10) hyperkalaemia (identified as serum potassium ≥ 5.0mEq/L) and (11) anaemia (defined as Hb < 11g/dl)].

Statistical significance recognized as *P* < .050 and denoted by ^a^

## 4 DISCUSSION

Low patient activation, the knowledge, skills and confidence a person has in managing their own health are associated with poor outcomes although there is limited research in CKD. Comparable to that cited in studies across other countries and LTCs,^8^, ^30^, ^31^ in our cohort, the mean PAM‐13 score was 55.1. We found that 26% of patients had Level 1 activation (least activated), signifying individuals tend to be passive and feel overwhelmed by managing their own health. In contrast, 13% of patients had the highest level of activation (Level 4). For context, in a sample of 305 Australian non‐dialysis CKD patients, Zimbudzi et al ^19^ reported a mean score of 57.6 and previous research has reported that between 10%‐22% of kidney patients fall into Level 1 with around 17 to 34% in Level 4.^15^, ^19^, ^21^ The median/mean level has been reported as between 2^32^ and 3.^14^, ^16^ A previous study by Hamilton et al,^23^ like our sample, found 26% of patients were in Level 1, and data from the UKRR revealed that across all stages 25% were in Level 1 and 17% were in Level 4.^12^ Nevertheless, comparisons between studies, especially those internationally, are difficult due to the heterogeneous populations included. Overall, the high number of patients with low activation and comparative PAM‐13 scores with those with advanced disease, such as dialysis, suggests our cohort of mostly mild to moderate CKD is characterized by poor patient activation.

Cardiovascular disease remains a leading cause of mortality and morbidity in CKD.^24^ We found patients with low activation had a greater number of CVD risk factors. A key CVD risk factor is age. The evidence between age and patient activation is inconsistent, although it has been hypothesized that younger patients may have poorer coping strategies which lead to low activation.^33^ Magnezi et al found that 25 Israeli participants aged 20‐29 with unknown kidney disease had lower activation levels compared with older patients.^22^ Other studies in different populations have shown no direct relationship between patient activation and age,^34^, ^35^, ^36^ although this may be due to differences in context (e.g. disease population, location). We found that older age *was* independently associated with low activation. Similar findings have been reported in diabetes^8^ and other chronic diseases,^4^, ^7^, ^8^ and our data support research in CKD by Van Bulk et al^32^ in dialysis patients and Zimbudzi et al^19^ in non‐dialysis CKD. Data from the UKRR^12^ found those aged 25‐44 and those who received transplant had the highest activation. There are many explanations for this, but it is likely older individuals have more complex health‐care needs (e.g. polypharmacy, functional limitations) and find self‐management behaviours more difficult. Consequently, interventions designed to aid self‐management, as well as activation, especially for the needs of older people are needed.

Low activated patients had a greater number of comorbidities. Study in older adults^4^ and cancer survivors^38^ has shown comorbidity is an important factor in patient activation. These findings are somewhat expected given that managing multiple LTCs likely results in an inability to cope.^39^, ^40^ We found that low activated patients were more likely to have diabetes, a key CVD risk factor. Diabetes is characterized by a large self‐management component and whilst having an additional LTC may increase the burden of self‐management, research by Zimbudzi et al^20^ found *higher* patient activation among patients *with* both diabetes *and* CKD. The authors attributed this to a focus on the skills accompanying diabetes self‐management. Similar findings have been shown in other LTCs; for example, Korpershoek et al^41^ found less comorbidities were associated with a lower activation in COPD. Whilst more studies are required, it appears that the nature of LTCs may influence self‐management behaviour.

We found that kidney function was lower in those with low activation and that PAM‐13 scores declined with disease progression. This observation supports work by Johnson et al^15^ who found patients with Stage 5 CKD had the lowest activation levels. Those with advanced disease have more complex health‐care needs than those with earlier stages and may find self‐management difficult. We identified that a significant proportion (42%) those with mild disease (Stage 1‐2) had low activation level. This suggests interventions in this group may confer positive benefits and may be more easily implemented in those with lower disease burden. Low activated patients had lower haemoglobin levels, and anaemia was present in approximately double those with low activation. Low haemoglobin is associated with fatigue, and it may be that these patients find self‐management tasks, like exercise, difficult. However, the UKRR data showed no association between patient activation and calcium, phosphorus, or haemoglobin, and in our data, the difference in haemoglobin between those with high/low activation was 5 g/L and should be interpreted with caution. Socioeconomic status is often considered to be an important factor in health‐care engagement. Studies have shown patient activation is only moderately correlated with SES^41^ and that education and income account for only <5 to 6% of the variation in patient activation.^9^ In contrast to what we expected, in a multivariate model, patients with low activation had a higher IMD, indicative of *greater* SES. However, differences were small and no difference in IMD was observed between the low and high activation groups.

We found patients with low activation had lower cardiorespiratory fitness and HRQOL. This supports a plethora of research in older adults and LTCs.^4^, ^7^, ^8^, ^9^ In CKD, data from the UKRR^12^ found those who reported better HRQOL had higher activation levels. Zimbudzi et al^20^ found low activated patients had a higher burden of kidney disease on the KDQOL‐36 questionnaire, including lower PCS and MCS. This suggests addressing mental and physical health issues may be important for enhancing patient activation and outcomes.^20^ Whilst cardiorespiratory fitness was estimated from the DASI and may indicate an inability to complete ADLs, it may be those with functional limitations find self‐management behaviours difficult.^4^ Furthermore, the inability to self‐manage may further exacerbate these functional limitations.

Contrary to what one may expect, we found no difference in fruit and vegetable intake between those with high and low activation. Despite healthy eating being mentioned in two questions in the PAM‐13, there is limited research investigating the role of patient activation on dietary intake. One previous study found that intervention‐derived increases in activation failed to change participants self‐reported adherence to a low‐fat diet.^42^ Our findings may be explained by the choice of self‐reported FFQ, although it may be the PAM‐13 is insensitive to detect such differences given that healthy eating is combined with other lifestyle behaviours (e.g. exercising) in each question.

We found the variables included explained 27% of the PAM‐13 score. The remaining variance may be explained by other factors influencing activation for self‐management, for example self‐efficacy, knowledge or the support from health‐care professionals. Previous research has shown greater activation is associated with greater knowledge of condition.^43^ The PAM‐13 includes items focussing on self‐efficacy, and whilst self‐efficacy measurement was not included in this study, in Social Cognitive Theory self‐efficacy is an important factor in self‐management skills and behavioural change.^44^

### 4.1 Strengths and limitations

Our study has several important strengths including the comprehensive range of biological and non‐biological variables analysed in a large population. The study was conducted across multiple sites from both secondary and primary care increasing the generalizability of our results. We used validated instruments for measuring HRQOL and patient activation. The limitations include the cross‐sectional design which does not allow assessment of temporal effects or the potential for reverse causality. Longitudinal studies are needed to better understand the effects over time of factors influencing patient activation. The use of self‐reported questionnaires may introduce misclassification due to socially desirable responses. Whilst the PAM‐13 is widely used in LTCs, it is limited by its self‐assessment of a patient's *perceived* ability to manage their own care, rather than the direct measurement of self‐management behaviour itself. Furthermore, patient activation in the setting of kidney disease may require knowledge and skills that are CKD‐specific; whether the PAM‐13 is an appropriate measure in kidney disease is unknown.^11^

### 4.2 Clinical recommendations

Interest in the PAM has been growing in nephrology, and further knowledge of characteristics associated with activation for self‐management is needed for the development of effective interventions. Based on our results, specific attention for any intervention should be paid to older patients with advancing CKD and multimorbidity. We did not identify easily ‘modifiable’ factors associated with patient activation, although this does not discount the role of interventions designed to improve factors such as knowledge and awareness, or access to social support. Further evidence is needed to define the role of patient activation as a mediator or moderator of clinical outcomes. For example, does improving a patient's activation help them better manage their condition which ultimately leads to other effects (e.g. improved HRQOL or a reduction in cost)? Or does activation moderate the benefit that patients report from other interventions (e.g. a low‐intensity web‐based self‐management intervention may demonstrate good outcomes with highly activated patients but may be ineffective in those with lower activation where more intensive intervention is needed).^4^ In kidney disease, how to maintain or remediate decline in patient activation is unknown.^11^ It is important to note that whilst in England, UK, the licence cost associated with the PAM‐13 is funded by NHS England and Improvement as part of a national agreement, this may not be the same in other health‐care organizations across the world.

## 5 CONCLUSION

This study showed only a minority of CKD patients are activated for self‐management, indicating a great possibility for improvement in self‐management and health outcomes. Measuring patient activation now forms part of the CKD management framework in the UK and in other countries across the world (e.g. the Kidney Care First and the Comprehensive Kidney Care Contracting models in the USA^11^). However, whilst including patient activation as a quality metric has the potential to target individuals at greatest risk,^1^ additional evidence is needed to better understand the role of patient activation on patients living with kidney disease.

## CONFLICT OF INTEREST

The author(s) declared no potential conflicts of interest with respect to the research, authorship and/or publication of this article. The authors declare that they have no competing interests.

## AUTHOR CONTRIBUTIONS

Conceptualization: TJW, KM, CJL, ACS; Methodology – data acquisition: KM, JP, TJW; Formal analysis and investigation: TJW; Writing ‐ original draft preparation: TJW; Writing ‐ review and editing: TJW, KM, CJL, JP, ACS; Funding acquisition: ACS; Supervision: ACS, TJW. All authors have full access to all the data in the study and takes responsibility for the integrity of the data and the accuracy of the data analysis. All authors gave final approval.

## Supporting information

Supplementary Material

## Data Availability

The data that support the findings of this study are available from the corresponding author upon reasonable request.
