# Supplementary material for: Determinants of patient activation and its association with cardiovascular disease risk in chronic kidney disease: A cross‐sectional study
Source: Health Expect. 2021 Apr 9;24(3):843–52. doi: 10.1111/hex.13225 (PMC8235879; doi:10.1111/hex.13225)
Supplement: Supplementary file 1 — Supplementary Material [file HEX-24-843-s001.docx]

**Supplementary material**

**Supplementary material 1. Recruiting trusts in UK**

| **1** | Cambridge University Hospitals | **9** | Cambridgeshire and Peterborough NHS Foundation Trust |
| --- | --- | --- | --- |
| **2** | Basildon and Thurrock University Hospitals | **10** | University Hospitals Plymouth NHS Trust |
| **3** | East Kent Hospitals University NHS Foundation Trust | **11** | Lancashire Teaching Hospitals NHS Trust |
| **4** | Royal Cornwall Hospitals NHS Trust | **12** | Sheffield Teaching Hospitals NHS Foundation Trust |
| **5** | Royal Devon & Exeter NHS Foundation Trust | **13** | University Hospital Southampton NHS Foundation Trust |
| **6** | East and North Hertfordshire NHS Trust | **14** | West Suffolk NHS Foundation Trust |
| **7** | University Hospitals Of Leicester NHS Trust |  |  |
| **8** | Mid Essex Hospital Services NHS Trust |  |  |

**Supplementary material 2. Patient Activation Measure score and number with low activation stratified by Index of Multiple Deprivation**

| **IMD decile** | **N** | **Mean (SD)** | **Adjusted mean‡** | **Low activation, n (%)** |
| --- | --- | --- | --- | --- |
| 1 (most deprived) | 34 | 53.5 (15.2) | 53.0 | 23 (66%) |
| 2 | 43 | 57.6 (14.1) | 56.3 | 18 (42%) |
| 3 | 63 | 54.7 (13.9) | 55.3 | 42 (66%) |
| 4 | 78 | 56.4 (12.6) | 56.8 | 46 (58%) |
| 5 | 64 | 56.1 (16.5) | 55.8 | 41 (63%) |
| 6 | 87 | 56.7 (15.8) | 56.2 | 46 (52%) |
| 7 | 98 | 54.1 (14.3) | 54.6 | 66 (65%) |
| 8 | 80 | 55.6 (12.6) | 55.6 | 46 (58%) |
| 9 | 75 | 53.8 (12.4) | 53.9 | 46 (61%) |
| 10 (least deprived) | 74 | 50.2 (14.6) | 50.6 | 53 (71%) |
| *Note.*  *Data presented as mean and standard deviation (SD) unless otherwise stated*  *Low activation defined as those in PAM level 1 or 2*  *IMD = Index of Multiple Deprivation*  *‡Estimated marginal means adjusted for age, eGFR, ethnicity* | | | | |

**Supplementary material 3. Odds of having low activation across CVD risk factors**

| **CVD risk factors** | **OR** | **Lower CI** | **Upper CI** | **P** |
| --- | --- | --- | --- | --- |
| Older age, >71 years old | 3.295 | 1.980 | 5.484 | <.001* |
| Male sex | .852 | .521 | 1.392 | .522 |
| Current smoker | 1.296 | .454 | 3.694 | .628 |
| Excessive alcohol, >14.3g/d | 1.258 | .560 | 2.830 | .578 |
| Obesity, BMI ≥30kg/h^2^ | 1.281 | .747 | 2.197 | .368 |
| Hypertension, present‡ | .793 | .489 | 1.286 | .347 |
| Diabetes, present | 1.735 | 1.003 | 3.000 | .049* |
| Anemia, Hb <11g/dl | 1.472 | .827 | 2.622 | .189 |
| Hyperkalemia, ≥5.0mEq/L | .822 | .494 | 1.368 | .450 |
| *Note.*  *Due to the large number of missing data, CRP, dyslipidemia, to leave a sample of 340. Analysis adjusted for eGFR* | | | | |
